# Supplementary material for: Saccharomyces boulardii (CNCM I-745) improves intestinal damage in sepsis by remodeling intestinal flora structure
Source: Front Cell Infect Microbiol. 2025 Jul 31;15:1602792. doi: 10.3389/fcimb.2025.1602792 (PMC12350329; doi:10.3389/fcimb.2025.1602792)
Supplement: Supplementary file 1 [file DataSheet1.zip › summary/summary/8_Advanced_analyse/2_PICRUSt2_result/KEGG/Group.BW0_vs_AW0/PICRUSt2_KEGG_level2_Group.BW0_vs_AW0_diff.pdf]

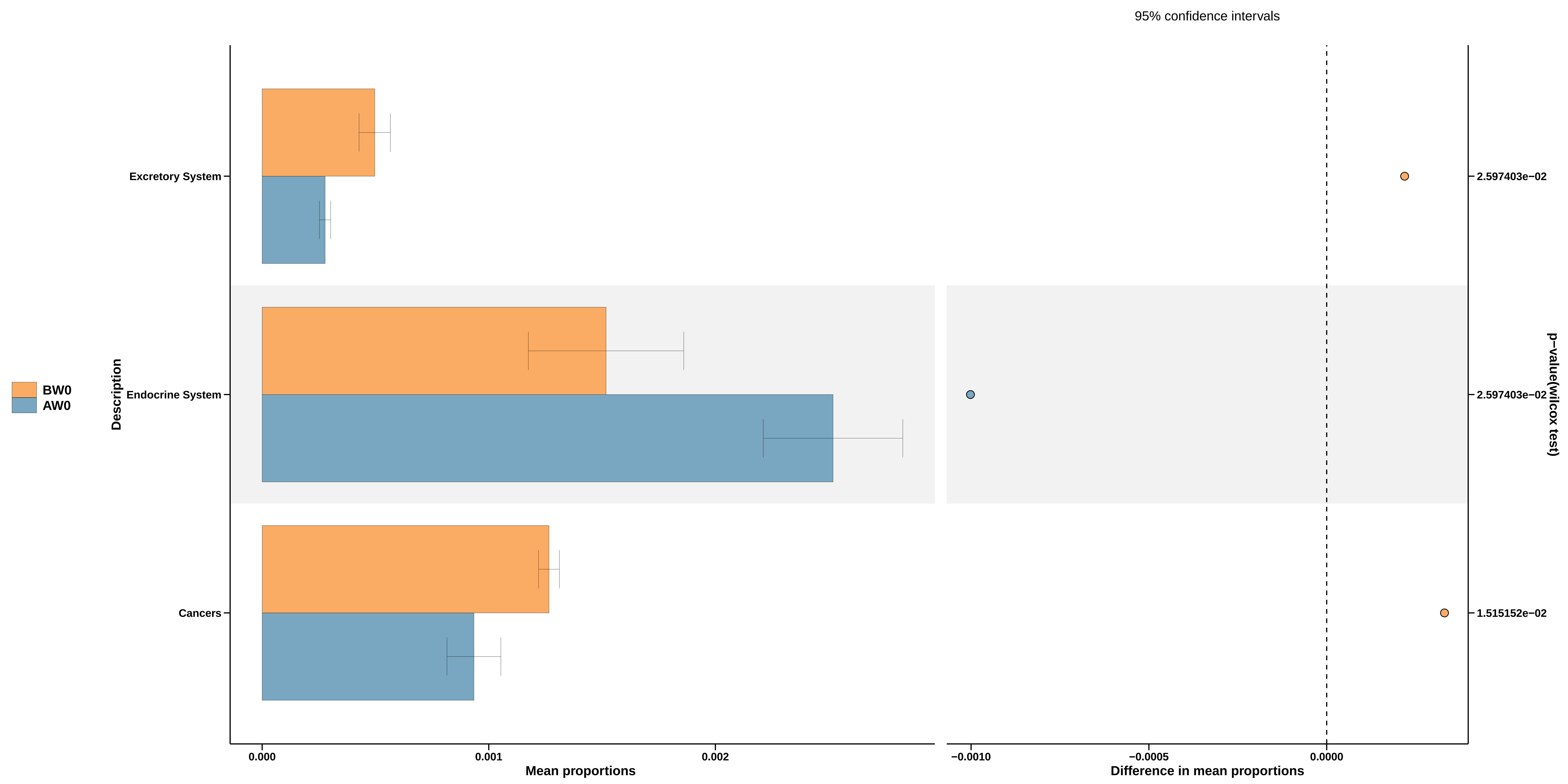
95% confidence intervals
BW0
AW0
Description
Excretory System
Endocrine System
Cancers
0.000
0.001
0.002
Mean proportions
−0.0010
−0.0005
0.0000
Difference in mean proportions
2.597403e−02
2.597403e−02
1.515152e−02
p−value(wilcox test)
